# Postpartum Glycaemic Phenotype and Continuous Glucose Monitoring at 4–6 Years After Gestational Diabetes Defined by Different Diagnostic Criteria

**DOI:** 10.1155/jdr/7103883

**Published:** 2026-09-08

**Authors:** Ulrika Andersson-Hall, Emilia Kristiansson, Sebastian Åberg, Matilda Törnqvist, Kristina Wallenius, Verena Sengpiel, Agneta Holmäng

**Affiliations:** ^1^ Institute of Neuroscience and Physiology, Department of Physiology, Sahlgrenska Academy, University of Gothenburg, Gothenburg, Sweden, gu.se; ^2^ Bioscience Metabolism, Research and Early Development, Cardiovascular, Renal and Metabolism (CVRM), BioPharmaceuticals R&D, AstraZeneca, Gothenburg, Sweden, astrazenecaindia.com; ^3^ Department of Obstetrics and Gynecology, Sahlgrenska University Hospital, Gothenburg, Region Västra Götaland, Sweden, sahlgrenska.se; ^4^ Department of Obstetrics and Gynecology, Institute of Clinical Sciences, Sahlgrenska Academy, University of Gothenburg, Gothenburg, Sweden, gu.se

**Keywords:** continuous glucose monitoring, gestational diabetes, glycaemic variability, postpartum, prediabetes, Type 2 diabetes

## Abstract

**Aim:**

To characterise glycaemic outcomes 4–6 years postpartum according to gestational diabetes mellitus (GDM) diagnostic thresholds and evaluate whether continuous glucose monitoring (CGM) identifies differences not captured by conventional clinical measures.

**Methods:**

Women recruited before and after introduction of WHO GDM criteria were followed at 4 and 6 years postpartum. At each follow‐up, participants underwent oral glucose tolerance testing (OGTT), HbA1c measurement, body composition assessment and 14‐day CGM. Based on pregnancy OGTT results and the diagnostic criteria in use at the time of testing, women were classified as meeting EASD 1991 criteria (GDM_OLD_; higher glucose thresholds), meeting WHO 2013 criteria but not EASD 1991 without diagnosis and treatment (GDM_WHO_−) or meeting WHO 2013 criteria with diagnosis and treatment (GDM_WHO_+).

**Results:**

Women classified as GDM_OLD_ had higher fasting glucose at both follow‐ups compared with women meeting only WHO 2013 criteria, and showed greater hyperglycaemic exposure and glycaemic variability on CGM. Time above range, coefficient of variation and mean amplitude of glycaemic excursions were consistently higher in GDM_OLD_ group, and differences remained significant after adjustment for pregnancy glucose levels. Within the WHO‐defined groups, conventional clinical measures did not differ between women with and without a GDM diagnosis, whereas CGM detected modest differences in mean glucose and time above range over time. Approximately 50% of women meeting only WHO 2013 criteria had prediabetes. CGM metrics were strongly associated with OGTT‐derived glucose and HbA1c but only weakly with anthropometric and blood lipid measures. BMI and body fat percentage did not differ between groups.

**Conclusions:**

Long‐term glycaemic risk aligns with diagnostic thresholds used during pregnancy. Women diagnosed by older, higher thresholds exhibit a persistently less favourable glycaemic profile. Women meeting the lower WHO 2013 glucose thresholds represent a large group with a high prevalence of prediabetes, regardless of diagnosis during pregnancy. CGM detects modest differences in free‐living glycaemic patterns not apparent from conventional clinical measures.

## 1. Introduction

Gestational diabetes mellitus (GDM), defined as hyperglycaemia first detected during pregnancy, is associated not only with adverse obstetric outcomes but also with a substantially increased risk of future dysglycaemia and cardiovascular disease [1, 2]. Women with prior GDM have a sevenfold to tenfold higher risk of developing Type 2 diabetes (T2D) and a twofold higher risk of cardiovascular disease compared with women with normoglycaemic pregnancies.

Despite these well‐established risks, diagnostic thresholds for GDM remain debated. In 2013, the World Health Organization (WHO), following recommendations from the International Association of Diabetes and Pregnancy Study Groups (IADPSG), introduced lower glucose thresholds for GDM diagnosis [3, 4]. These criteria were based on associations between maternal glycaemia and adverse perinatal outcomes in the Hyperglycaemia and Adverse Pregnancy Outcome (HAPO) study rather than on future maternal metabolic risk [5]. Adoption of the WHO 2013 criteria increases the number of women diagnosed with GDM, but the long‐term implications for maternal glycaemic health remain uncertain [6, 7].

In Sweden, the Changing Diagnostic Criteria for Gestational Diabetes in Sweden (CDC4G) study evaluated the transition from the previous, higher EASD 1991 diagnostic thresholds to the WHO 2013 criteria [8]. CDC4G used a stepped‐wedge cluster‐randomised design in which participating maternity units introduced the WHO 2013 criteria at randomly allocated time points. Implementation increased the prevalence of GDM and improved maternal composite outcome but did not significantly reduce the occurrence of large‐for‐gestational‐age births [9]. At Sahlgrenska University Hospital, this design meant that women with glucose values meeting only the WHO 2013 thresholds received a GDM diagnosis and treatment after but not before implementation. This setting enables comparison of long‐term outcomes according to both the glucose thresholds met during pregnancy and whether a clinical diagnosis was made.

Postpartum surveillance after GDM relies primarily on intermittent clinical measures, including fasting plasma glucose, oral glucose tolerance tests (OGTT) and HbA1c [10]. In routine practice, follow‐up is inconsistent across settings and often relies on periodic fasting glucose and/or HbA1c rather than repeated OGTTs, with generally low adherence to recommended screening [11–13]. Early detection of dysglycaemia is critical, as timely diagnosis of T2D enables interventions to prevent or delay secondary complications [10]. However, these conventional measures provide only limited insight into free‐living glucose regulation and may fail to detect early abnormalities driven by impaired beta‐cell function and exaggerated postprandial excursions [14–16].

Continuous glucose monitoring (CGM) captures free‐living glucose exposure, time in range and glycaemic variability and has been proposed as a sensitive tool for identifying early dysglycaemia [14, 15, 17]. However, longitudinal CGM data beyond the early postpartum period in women with prior GDM and in high‐risk populations before the onset of overt diabetes remain limited.

In a previous 2‐year follow‐up of this cohort, we showed that women diagnosed according to older, higher glucose threshold criteria exhibited a more adverse glycaemic profile than women fulfilling WHO 2013 criteria, despite similar BMI at the 2‐year follow‐up [18]. Whether these differences persist and to what extent they are reflected by conventional testing versus CGM remains unknown.

The present study extends this work by examining long‐term glycaemic outcomes 4–6 years postpartum in relation to GDM diagnostic criteria. We compared women diagnosed according to older, higher glucose thresholds with those meeting WHO 2013 criteria and, within the WHO‐defined group, women with and without a clinical diagnosis and treatment. We further explored whether CGM provides complementary information on free‐living glucose regulation beyond conventional clinical measures and whether women identified only by the lower WHO 2013 criteria represent a clinically relevant high‐risk group.

## 2. Methods

### 2.1. Study Population

Participants were recruited from the ongoing PREvention of PostPartum (PREPP) diabetes study, which follows women who underwent pregnancy OGTT at Sahlgrenska University Hospital, Gothenburg during the preparation and data collection phase of the CDC4G trial [8, 18]. The PREPP study includes assessments at 2, 4 and 6 years postpartum. The present analyses are based on the 4‐ and 6‐year visits conducted between May 2021 and August 2025.

During the preparation and data collection phase for the CDC4G trial (September 2017–December 2018), all pregnant women living in the Gothenburg area, with risk factors for GDM (*n* = 2117) were referred for a 75‐g OGTT at Sahlgrenska University Hospital, Gothenburg, during gestational Weeks 25–28 [8]. The risk factors for OGTT referral were diabetic heredity, high nonfasting glucose at maternal clinic, BMI ≥ 35 kg/m^2^, prior pregnancy with GDM/macrosomia/large‐for‐gestational‐age or accelerated foetal growth and/or polyhydroamnios. High nonfasting capillary glucose was defined as ≥ 8 mmol/L. LGA was defined as birth weight ≥ +2 SD above the Swedish reference curve [19] and macrosomia as birth weight ≥ 4.5 kg.

Of the eligible women, 860 women fulfilled the WHO 2013 criteria for GDM and were subsequently invited to participate in the PREPP study. Invitations were sent by telephone, SMS, email and/or letter. Women who had experienced an additional pregnancy within 12 months prior to any of the follow‐up visits were excluded for that visit but were eligible for any of the other visits. Two women who were diagnosed with Type 1 diabetes and one woman who had several severe comorbidities were excluded from the present analysis.

**Figure 1:**
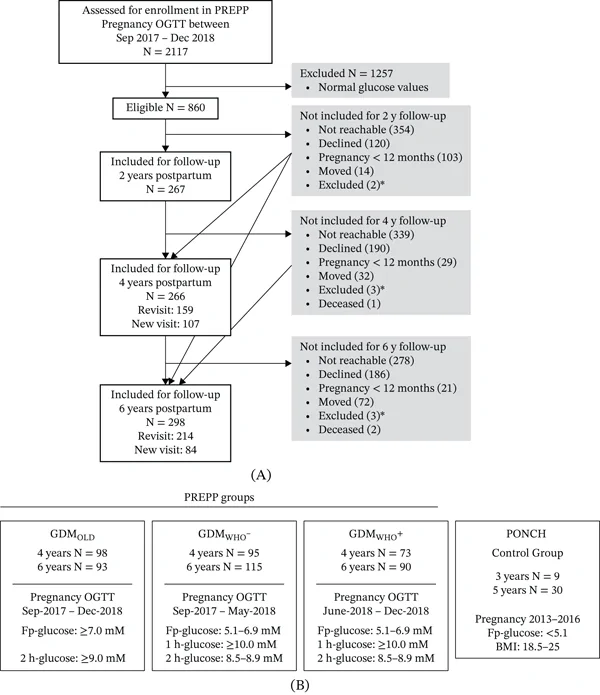
(A) PREPP study flow chart. (B) Classification of PREPP participants according to gestational diabetes diagnostic criteria, including the PONCH control group. Women in the GDM_OLD_ and GDM_WHO_+ groups received a GDM diagnosis and treatment, whereas women in the GDM_WHO_− group met WHO 2013 glucose thresholds but did not receive a diagnosis or treatment.  ^∗^Exclusion due to Type 1 diabetes or severe comorbidity.

At the 4‐year follow‐up, 266 women participated, of whom 159 were revisits from the 2‐year follow‐up [18] and the remaining attended their first study visit. At the 6‐year follow‐up, 298 women participated, including 214 revisits and 84 newly included participants. A detailed overview of participant inclusion and exclusion is provided in Figure 1.

In June 2018, Sahlgrenska University Hospital changed diagnostic criteria for GDM from the EASD 1991 criteria (fasting plasma glucose ≥ 7.0 mmol/L and/or 2‐h plasma glucose ≥ 9.0 mmol/L) to the WHO 2013 criteria (fasting plasma glucose ≥ 5.1 mmol/L, 1‐h plasma glucose ≥ 10.0 mmol/L and/or 2‐h plasma glucose ≥ 8.5 mmol/L). Based on glucose values at the pregnancy OGTT and the timing relative to the change in diagnostic criteria, eligible PREPP participants were stratified into three groups:

• GDM_OLD_: women whose glucose values met the higher EASD 1991 criteria and who therefore received a GDM diagnosis and treatment.
• GDM_WHO_−: women whose glucose values met WHO 2013 criteria (but not the older EASD 1991 criteria) and did not receive a GDM diagnosis or treatment because the OGTT was performed before the criteria change
• GDM_WHO_+: women whose glucose values met WHO 2013 criteria (but not the older EASD 1991 criteria) and who received a GDM diagnosis and treatment because the OGTT was performed after the criteria change.

When comparing GDM_OLD_ with women diagnosed according to WHO criteria only, the two WHO groups are combined into one group: GDM_WHO_.

In the Gothenburg region, women diagnosed with GDM received lifestyle counselling focusing on diet and physical activity. If glycaemic targets were not achieved, pharmacological treatment with metformin and/or insulin was initiated according to regional practice, in line with WHO recommendations. Foetal growth in women with GDM diagnosis was followed with abdominal ultrasound at least every 4th week. Women with pharmacological treatment were recommended induction of labour at 40 + 0 gestational weeks. Women in the GDM_WHO−_ group did not receive a GDM diagnosis and therefore did not receive structured treatment or specialised follow‐up during pregnancy.

After birth, women diagnosed with GDM were referred to primary care for follow‐up within a year, which typically included measurement of fasting plasma glucose and/or HbA1c. However, follow‐up was not organised within a structured postpartum programme, and routine postpartum OGTT was not systematically performed in this setting.

A control group from the Pregnancy Obesity Nutrition and Child Health (PONCH) cohort [20] was included to provide reference values for clinical variables. These women were normoglycaemic and of normal weight. Study visits were conducted at 3 and 5 years postpartum using the same protocol as in the PREPP study, except that no OGTT was performed. The 3‐year data were used as reference for PREPP 4‐year follow‐up, and the 5‐year data for the 6‐year follow‐up. No formal analysis was performed between the GDM groups and control group.

### 2.2. Study Visits

After 4 and 6 years of pregnancy, participants attended Sahlgrenska University Hospital after an overnight fast. Venous blood samples were collected for measurement of plasma glucose, HbA1c, insulin, C‐peptide, triglycerides, LDL cholesterol and HDL cholesterol. A 75‐g OGTT was offered to participants without previously diagnosed T2D whose fasting capillary blood glucose was < 8.0 mmol/L.

For the OGTT, venous glucose and insulin were obtained before glucose ingestion and after 30, 60, 90 and 120 min. Capillary glucose measurements were used as backup at 0 and 120 min when venous sampling was unsuccessful, or at all time points when peripheral venous catheterisation was unsuccessful (*n* = 9 at 4 years and *n* = 6 at 6 years). Venous samples were analysed at the Laboratory of Clinical Chemistry, Sahlgrenska University Hospital, which is certified according to ISO 15189:2007.

Body weight, height, waist and hip circumferences were measured at each visit. Body composition was assessed by air displacement plethysmography (BOD POD; Software Version 5.4.0, COSMED), providing estimates of fat mass and fat‐free mass.

Participants were also asked to wear a CGM device (Freestyle Libre Pro iQ, Abbott Diabetes Care Ltd) for 14 consecutive days. Interstitial glucose was recorded every 15 min, providing measures of mean glucose, time in range and glucose variability. If the monitor fell off before the 14 days were completed, the participants were offered a new monitor.

### 2.3. Data Processing

OGTT data were used to derive indices of insulin resistance and *β*‐cell function. HOMA‐IR was calculated from fasting plasma glucose and fasting insulin as (fasting glucose × fasting insulin)/22.5, and HOMA‐*β* as (20 × fasting insulin)/(fasting glucose 3.5), as previously described [21]. HOMA‐IR primarily reflects hepatic insulin resistance, whereas HOMA‐*β* provides an estimate of basal *β*‐cell function. Whole‐body insulin sensitivity was assessed using the Matsuda index, calculated from fasting and mean glucose and insulin concentrations during the OGTT [22]. Early‐phase insulin secretion was estimated using the insulinogenic index (*Δ* insulin 30–0 min/*Δ* glucose 30–0 min) [23]. Glucose and insulin area under the curve (AUC) during the OGTT were calculated using the trapezoidal rule. When venous glucose samples were unavailable, capillary glucose values were converted to venous equivalents using established conversion formulas [24].

Based on OGTT results or previously reported T2D diagnosis, participants were classified into glucose tolerance categories: normal glucose tolerance (NGT), prediabetes or T2D. Prediabetes was defined as fasting plasma glucose 5.6–6.9 mmol/L and/or 1‐h plasma glucose 8.6–11.5 mmol/L and/or 2‐h plasma glucose 7.8–11.0 mmol/L. T2D was defined as fasting plasma glucose ≥ 7.0 mmol/L and/or 1‐h plasma glucose ≥ 11.6 mmol/L and/or 2‐h plasma glucose ≥ 11.1 mmol/L [25].

CGM data were used to derive measures of glycaemic variability, including coefficient of variation and the mean amplitude of glycaemic excursions (MAGE) [14]. Time in range was defined as interstitial glucose 3.9–7.8 mmol/L, reflecting a proposed target range for individuals with prediabetes (sometimes referred to as time in tight range) [14, 26]. Data from the first CGM day were excluded from analyses to allow for sensor stabilisation in line with manufacturer′s recommendations.

### 2.4. Statistical Analysis

Baseline group differences were analysed using Student′s *t*‐test for continuous variables and *χ* ^2^ tests or Fisher′s exact test for categorical variables. Group differences at 4 and 6 years postpartum were analysed using linear mixed‐effects models including group, time and group × time interaction terms and adjusted for reason for OGTT, BMI at first antenatal visit, ethnicity and maternal age at birth. Additional analyses were further adjusted for pregnancy glucose values (fasting and 2‐h). Corresponding unadjusted *p* values are presented in supporting material.

Analyses were conducted in two predefined comparison steps. First, women in GDM_OLD_ were compared with women in GDM_WHO_ (including both GDM_WHO_+ and GDM_WHO_−) to assess differences according to diagnostic threshold. Second, within GDM_WHO_, outcomes were compared between GDM_WHO_+ and GDM_WHO_−.

Correlation patterns between clinical glucose measures and CGM‐derived metrics were similar at 4 and 6 years postpartum, supporting the use of pooled analyses. To increase statistical power, these associations were therefore examined using pooled data from the 4‐ and 6‐year follow‐ups, with time point–specific results presented in the supporting material. Spearman correlation coefficients were calculated, with standard errors adjusted for repeated observations using cluster‐robust variance estimation at the participant level.

All statistical analyses were performed using R (Version 4.5.2). Linear mixed‐effects models were fitted using the lme4 and lmerTest packages. For pooled correlation analyses, cluster‐robust standard errors were estimated using the sandwich and lmtest packages.

### 2.5. Ethics

Ethical approval for PREPP was granted by the Regional Ethical Review Board in Gothenburg (Dnr 2019‐01300 and 2021‐02618), whereas PONCH was approved under Dnr 402‐08. All participants provided written informed consent before their first study assessment. Participation was voluntary, and women could decline individual study procedures or withdraw from the study without consequence.

## 3. Results

### 3.1. Participant Characteristics and Pregnancy Metabolic Profile

Baseline characteristics during pregnancy are presented in Table 1 for women in the GDM_WHO_−, GDM_WHO_+ and GDM_OLD_ groups, together with a normoglycaemic, normal‐weight control group for reference. Women diagnosed according to older criteria were slightly older when giving birth than women classified by WHO criteria. As per definition, pregnancy OGTT profiles reflected the diagnostic definitions: The GDM_OLD_ group exhibited markedly higher 2‐h glucose concentrations, whereas fasting glucose values were similar across groups. Ethnic distribution and indication for OGTT differed between GDM_OLD_ and GDM_WHO_ groups, where women in the GDM_OLD_ group more often were of non‐European ethnicity and were called for a pregnancy OGTT due to high random glucose values at an antenatal visit. Use of glucose‐lowering treatment during pregnancy was more common in the GDM_OLD_ group. When comparing the two GDM_WHO_ groups, fasting glucose was slightly higher in GDM_WHO_− compared with GDM_WHO_+ at pregnancy OGTT. Additionally, there was a significant difference in prescribed medication since only the GDM_WHO_+ group got a diagnosis and treatment.

**Table 1:** Background and pregnancy characteristics.

	Control (*n* = 32)	GDM_OLD_ (*n* = 129)	GDM_WHO_− (*n* = 136)	GDM_WHO_+ (*n* = 112)	*p*GDM_OLD_ versus GDM_WHO_	*p*GDM_WHO_+ versus GDM_WHO_−
Age at birth (years)	31.1 ± 3.8	34.2 ± 4.9	33.5 ± 4.5	33.1 ± 4.5	0.088	0.516
BMI (kg/m^2^)∗	22.3 ± 1.6	28.5 ± 6.1	28.6 ± 6.2	28.4 ± 6.1	0.998	0.817
Gestational weight gain (kg)	11.5 ± 2.3	10.0 ± 6.0	14.0 ± 6.3	10.9 ± 5.9	< 0.001	< 0.001
Fasting glucose, pregnancy (mmol/L)	4.5 ± 0.3	5.4 ± 0.9	5.4 ± 0.4	5.2 ± 0.4	0.090	< 0.001
2 h glucose, pregnancy (mmol/L)	—	10.0 ± 1.2	7.2 ± 1.1	7.3 ± 1.2	< 0.001	0.620
Ethnicity (% Nordic/other‐European/non‐European)	97/3/0	37/8/55	57/5/38	52/10/38	0.005	0.348
Family history of diabetes (%)	—	73	74	71	1.000	0.689
Parity (0/1/≥ 2)	58/32/10	38/35/27	44/40/16	38/38/24	0.269	0.287
Reason for OGTT (% risk factors/random glucose/GDM or LGA history/other)	—	51/40/7/2	63/22/14/1	68/23/7/2	0.003	0.321
Metformin treatment, pregnancy (%)	0	11	0	4	< 0.001	0.018
Insulin treatment, pregnancy (%)	0	5	0	4	0.098	0.041
Both metformin and insulin treatment, pregnancy (%)	0	6	0	3	0.010	0.092

*Note:* Women were classified as GDM_WHO_− (WHO 2013 criteria with no diagnosis), GDM_WHO_+ (WHO 2013 criteria) or GDM_OLD_ (EASD 1991 criteria). A normoglycaemic, normal‐weight control group is shown for reference. Continuous variables are presented as mean ± SD and compared using Student′s *t* test; categorical variables are presented as percentages and compared using *χ* ^2^ or Fisher′s exact tests, as appropriate. *p* GDM_OLD_ versus GDM_WHO_ signifies comparison between GDM_OLD_ and pooled GDM_WHO_ groups. *p* GDM_WHO_+ versus GDM_WHO_− signifies comparison between the GDM_WHO_ groups. Asterisk “ ^∗^” denotes BMI at first antenatal visit.

### 3.2. WHO Versus Old Criteria: Long‐Term Glycaemic and Metabolic Phenotype From 4 to 6 Years Postpartum

We first compared women in the GDM_OLD_ group with women in the GDM_WHO_ group (including both GDM_WHO_+ and GDM_WHO_−). Across follow‐up visits, women in the GDM_OLD_ group displayed a less favourable glycaemic profile compared with the GDM_WHO_ group despite largely similar levels of adiposity (Tables S1 and 2).

**Table 2:** Outcomes at 4 and 6 years postpartum by GDM diagnostic criteria (GDM_OLD_ vs. GDM_WHO_), with normoglycaemic controls.

	Time	Control	GDM_OLD_	GDM_WHO_	*p*group	Longitudinal model *p*		
						Group	Time	Interaction
*n*	4y/6y	9/30	98/93	168/205				
BMI (kg/m^2^)	4y	22.0 ± 1.6	28.3 ± 6.5	29.3 ± 7.2	0.006	0.018	0.423	0.201
	6y	22.4 ± 1.9	28.4 ± 6.6	29.1 ± 6.9	0.161			
Body fat %	4y	23.6 ± 6.0	39.0 ± 9.7	39.4 ± 9.8	0.303	0.557	0.484	0.456
	6y	26.6 ± 6.1	39.2 ± 9.2	39.3 ± 9.5	0.753			
Waist (cm)	4y	71.8 ± 3.9	92.7 ± 13.9	95.0 ± 16.0	0.071	0.164	0.331	0.155
	6y	73.6 ± 5.1	92.9 ± 14.8	94.3 ± 16.3	0.768			
HbA1c (mmol/mol)	4y	32.2 ± 1.9	35.9 ± 5.8	33.5 ± 2.9	< 0.001	< 0.001	0.045	0.676
	6y	31.4 ± 2.9	35.9 ± 4.4	33.8 ± 3.6	< 0.001			
Fasting glucose (mmol/L)	4y	5.1 ± 0.52	5.8 ± 0.8	5.5 ± 0.5	< 0.001	< 0.001	0.259	0.895
	6y	5.2 ± 0.38	5.7 ± 0.9	5.5 ± 0.5	< 0.001			
2 h glucose (mmol/L)	4y	—	7.2 ± 2.6	5.8 ± 1.3	< 0.001	< 0.001	0.333	0.499
	6y	—	6.7 ± 2.2	5.7 ± 1.4	< 0.001			
HOMA‐IR	4y	1.2 ± 0.59	2.3 ± 2.0	2.1 ± 1.3	0.402	0.626	0.064	0.915
	6y	0.97 ± 0.38	2.0 ± 1.5	1.8 ± 1.4	0.469			
*CGM metrics*		—						
Mean glucose (mmol/L)	4y	—	5.9 ± 0.9	5.5 ± 0.5	0.001	0.001	0.411	0.212
	6y	—	5.7 ± 0.6	5.5 ± 0.4	0.043			
Time in range (%)	4y	—	89.7 ± 14.1	96.4 ± 3.8	< 0.001	< 0.001	0.252	0.392
	6y	—	92.8 ± 7.2	96.7 ± 4.0	< 0.001			
Time below range (%)	4y	—	1.7 ± 2.8	1.7 ± 2.8	0.723	0.910	0.517	0.923
	6y	—	1.4 ± 1.8	1.4 ± 2.3	0.801			
Time above range (%)	4y	—	8.6 ± 14.5	1.9 ± 3.1	< 0.001	< 0.001	0.380	0.415
	6y	—	5.8 ± 7.3	1.9 ± 3.1	< 0.001			
CV glucose	4y	—	17.5 ± 4.0	13.9 ± 2.9	< 0.001	< 0.001	0.615	0.541
	6y	—	17.5 ± 4.0	14.1 ± 3.5	< 0.001			
MAGE (mmol/L)	4y	—	1.3 ± 0.4	1.1 ± 0.2	< 0.001	< 0.001	0.575	0.474
	6y	—	1.3 ± 0.3	1.1 ± 0.3	< 0.001			

*Note:* Values are presented as means ± SD. GDM_WHO_ includes all women fulfilling WHO 2013 glucose criteria, irrespective of clinical diagnosis. The control group is shown for reference only and was not included in statistical comparisons. Group differences were analysed using linear mixed‐effects models with participant‐specific random intercepts, adjusted for reason for OGTT, BMI at first antenatal visit, ethnicity and maternal age at birth. *p* values at each time point represent adjusted comparisons between GDM_OLD_ and GDM_WHO_ at 4 and 6 years postpartum, derived from this model. Longitudinal *p* values represent overall group, time and interaction effects. Time in range was defined as interstitial glucose 3.9–7.8 mmol/L.

Abbreviations: CV, coefficient of variation; HOMA‐IR, homeostatic model assessment of insulin resistance; MAGE, mean amplitude of glycaemic excursions.

**Figure 2:**
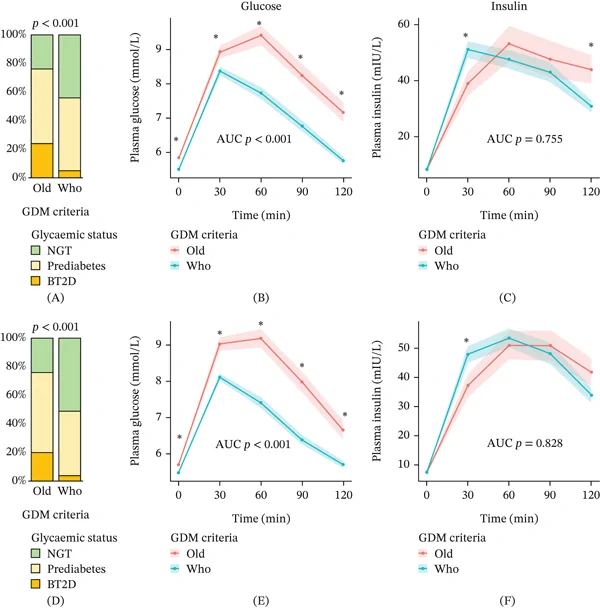
OGTT results in women meeting older diagnostic criteria (GDM_OLD_) compared with women fulfilling WHO 2013 criteria (GDM_WHO_). (A,D) Distribution of glucose tolerance categories at 4 and 6 years postpartum. (B–C) OGTT glucose and insulin curves at 4 years postpartum. (E–F) OGTT glucose and insulin curves at 6 years postpartum. Values are presented as mean ± SE. Group differences in glucose tolerance distribution were assessed using *χ* ^2^ tests. Overall differences in glucose curves were assessed using glucose AUC comparisons. Asterisks indicate significant between‐group differences at specific OGTT time points.

The prevalence of dysglycaemia (prediabetes or T2D) was high in both diagnostic groups (Figure 2). Women in the GDM_OLD_ group had a higher proportion of T2D at both follow‐ups (24% vs. 5% at 4 years and 20% vs. 4% at 6 years), whereas prediabetes was common in both groups (52%–56% in GDM_OLD_ and 45%–51% in GDM_WHO_). A relatively low proportion of T2D cases had been diagnosed in routine clinical follow‐up prior to the study visit (7 of 24 at 4 years and 10 of 19 at 6 years in GDM_OLD_, and 1 of 9 and 3 of 8 in GDM_WHO_). Across follow‐up, fasting glucose was approximately 0.2–0.3 mmol/L higher, 2‐h glucose around 1 mmol/L higher and HbA1c approximately 2 mmol/mol higher in GDM_OLD_ than GDM_WHO_ (Table 2). These differences were stable over time, with no strong evidence of group‐by‐time interactions, indicating a persistent risk gradient rather than divergent trajectories. Differences in glucose remained significant, even after further adjustments for different pregnancy glucose levels, whereas the difference in HbA1c was attenuated and no longer statistically significant (Table S1).

BMI differed modestly between groups at 4 years, with slightly higher values in GDM_WHO_; however, no consistent differences were observed in body composition, insulin resistance or other cardiometabolic markers (Tables S1 and 2). Weight retention from early pregnancy to follow‐up differed between groups, with greater weight reduction in GDM_OLD_ (Table S1), despite lower gestational weight gain (Table 1). Available fasting glycaemic, anthropometric and body composition measures were higher in both GDM groups than in the control group, which comprised women with normoglycaemic and normal‐weight pregnancies.

CGM‐derived outcomes consistently differentiated the GDM_OLD_ and pooled GDM_WHO_ groups across follow‐up (Table 2). Women in the GDM_OLD_ group had higher time above range (approximately 6%–9% vs. 2%) and exhibited greater glycaemic variability, as reflected by higher coefficient of variation and MAGE, at both 4 and 6 years postpartum. Differences in time in/above range and CV remained significant when adjusted for differences in pregnancy glucose levels (Table S1). Differences in mean CGM glucose were significant at 4 years but not at 6 years; after adjustments for pregnancy glucose levels, no significant between‐group differences in mean glucose remained.

OGTT profiles were consistent with these findings. Women in the GDM_OLD_ group exhibited much higher postload glucose concentrations and somewhat higher insulin responses compared with GDM_WHO_ (Figure 2), suggesting differences in postprandial glucose regulation.

Together, these findings indicate that women diagnosed according to older criteria exhibit a distinct and persistently less favourable glycaemic phenotype several years postpartum compared with women meeting WHO 2013 glucose thresholds.

### 3.3. GDM_WHO_− Versus GDM_WHO_+: Clinical and CGM Outcomes at 4 and 6 Years Postpartum

As a second step, we compared GDM_WHO_− with GDM_WHO_+. Conventional clinical measures of glucose tolerance did not differ between groups at either follow‐up (Table 3). HbA1c, HOMA‐IR, fasting and OGTT plasma glucose were similar at both 4 and 6 years postpartum. Likewise, BMI, waist circumference, blood lipids, blood pressure and most anthropometric measures did not differ between groups (Tables S2 and 3). Despite a larger gestational weight gain in GDM_WHO_− than GDM_WHO_+ (Table 1), there was no difference in weight retention from early pregnancy to postpartum follow‐up (Table S2). Body fat percentage was slightly lower in GDM_WHO_− at 4 years postpartum but not at 6 years. Longitudinal models showed no evidence of differential change over time for other clinical measures.

**Table 3:** Clinical and CGM outcomes at 4 and 6 years postpartum in women meeting WHO 2013 criteria (GDM_WHO_− vs. GDM_WHO_+).

	Time	GDM_WHO_−	GDM_WHO_+	*p* group	Longitudinal model *p*		
					Group	Time	Interaction
*N*	4y/6y	95/115	73/90				
BMI (kg/m^2^)	4y	29.3 ± 7.6	29.2 ± 6.7	0.282	0.451	0.465	0.241
	6y	29.1 ± 7.1	29.0 ± 6.6	0.837			
Body fat %	4y	38.9 ± 10.1	40.0 ± 9.4	0.047	0.059	0.100	0.035
	6y	39.3 ± 10.1	39.2 ± 8.8	0.917			
Waist (cm)	4y	96.1 ± 16.7	93.5 ± 15.0	0.559	0.777	0.426	0.507
	6y	94.4 ± 16.6	94.2 ± 16.0	0.920			
HbA1c (mmol/mol)	4y	33.5 ± 2.8	33.4 ± 3.0	0.977	0.612	0.048	0.351
	6y	33.8 ± 3.5	33.9 ± 3.7	0.570			
Fasting glucose (mmol/L)	4y	5.6 ± 0.5	5.4 ± 0.5	0.126	0.273	0.388	0.518
	6y	5.5 ± 0.5	5.4 ± 0.5	0.359			
2 h glucose (mmol/L)	4y	5.7 ± 1.2	5.9 ± 1.4	0.172	0.209	0.865	0.856
	6y	5.6 ± 1.6	5.8 ± 1.3	0.176			
HOMA‐IR	4y	2.0 ± 1.3	2.1 ± 1.3	0.663	0.519	0.076	0.273
	6y	1.9 ± 1.5	1.7 ± 1.3	0.395			
*CGM metrics*							
Mean glucose (mmol/L)	4y	5.6 ± 0.5	5.4 ± 0.5	0.001	< 0.001	< 0.001	< 0.001
	6y	5.5 ± 0.4	5.6 ± 0.5	0.006			
Time in range (%)	4y	96.6 ± 3.4	96.2 ± 4.2	0.577	0.007	0.251	0.105
	6y	97.4 ± 3.6	95.7 ± 4.3	0.002			
Time below range (%)	4y	1.4 ± 2.6	2.1 ± 3.2	0.108	0.125	0.603	0.632
	6y	1.2 ± 2.1	1.6 ± 2.6	0.225			
Time above range (%)	4y	1.9 ± 2.7	1.8 ± 3.6	0.555	0.008	0.038	0.011
	6y	1.4 ± 2.1	2.7 ± 3.9	0.003			
CV glucose	4y	13.5 ± 3.0	14.6 ± 2.6	0.046	0.035	0.658	0.748
	6y	13.8 ± 3.8	14.5 ± 3.0	0.053			
MAGE (mmol/L)	4y	1.0 ± 0.2	1.1 ± 0.2	0.723	0.034	0.161	0.130
	6y	1.0 ± 0.3	1.1 ± 0.2	0.011			

*Note:* Values are presented as means ± SD. GDM_WHO_− and GDM_WHO_+ include women fulfilling WHO 2013 glucose criteria without and with a clinical diagnosis and treatment during pregnancy, respectively. Group differences were analysed using linear mixed‐effects models with participant‐specific random intercepts, adjusted for reason for OGTT, BMI at first antenatal visit, ethnicity and maternal age at birth. *p* values at each time point represent group comparisons at 4 and 6 years postpartum, derived from this model. Longitudinal *p* values represent overall group, time and interaction effects. Time in range was defined as interstitial glucose 3.9–7.8 mmol/L.

Abbreviations: CV, coefficient of variation; HOMA‐IR, homeostatic model assessment of insulin resistance; MAGE, mean amplitude of glycaemic excursions.

CGM‐derived mean glucose, time in range and time above range showed modest differences between GDM_WHO_− and GDM_WHO_+ with significant group‐by‐time interactions for mean CGM glucose and time above range. At 4 years postpartum, mean CGM glucose was slightly higher in GDM_WHO_−, whereas at 6 years postpartum mean glucose and time above range were slightly higher in GDM_WHO_+. Absolute differences were small and remained within largely normoglycaemic ranges (Table 3).

Other CGM variability metrics also suggested modest differences between groups. CV glucose was higher in GDM_WHO_+ at 4 years and showed a similar trend at 6 years, whereas MAGE differed mainly at 6 years. Overall group effects were observed for both metrics, but these were attenuated after adjustment for pregnancy glucose (Table S2).

There were no differences between GDM_WHO_− and GDM_WHO_+ in glucose tolerance distribution (NGT/prediabetes/T2D) or glucose and insulin OGTT curves (Figure S1).

Taken together, these findings indicate that conventional clinical measures were largely similar between women above WHO cutoffs who did and did not receive a GDM diagnosis during pregnancy, whereas CGM detected modest differences in free‐living glycaemia.

### 3.4. Associations Between Clinical Glucose Measures and Free‐Living Glycaemia

**Figure 3:**
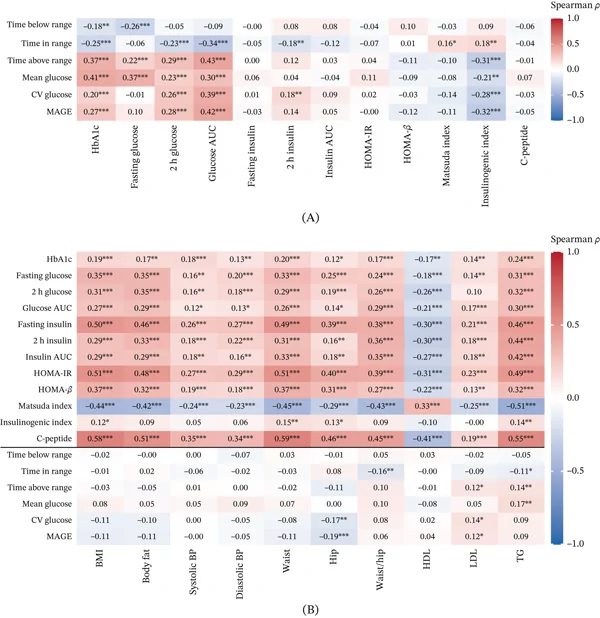
(A) Heatmap of associations between clinical glucose variables and CGM‐derived metrics. (B) Heatmap of associations between glucose variables (clinical and CGM‐derived) and other clinical measurements, including anthropometry, blood lipids and blood pressure. Data from the 4‐ and 6‐year follow‐ups were pooled (266 women at 4 years and 298 at 6 years postpartum). Pairwise sample sizes ranged from 400 (CGM vs. OGTT variables) to 561 (clinical variables only), depending on data availability. Associations were assessed using Spearman correlation coefficients with cluster‐robust standard errors to account for repeated measurements within individuals. Colour intensity represents Spearman′s *ρ*. *p* < 0.05, *p* < 0.01, *p* < 0.001. HOMA‐IR, homeostatic model assessment of insulin resistance; CV, coefficient of variation; MAGE, mean amplitude of glycaemic excursions.

To complement the group comparisons, we examined associations between CGM‐derived metrics and conventional clinical glucose measures using pooled data from the 4‐ and 6‐year follow‐ups (Figure 3). Correlation patterns were highly similar at both time points (Figures S2 and S3, showing 4‐ and 6‐year data separately), supporting the use of pooled analyses to increase statistical precision.

In this high‐risk cohort of women with prior GDM, CGM measures showed the strongest associations with OGTT‐derived glucose (2 h glucose and glucose AUC) and HbA1c (Figure 3A). In contrast, fasting glucose and insulin, indices of insulin resistance (HOMA‐IR and Matsuda index), OGTT insulin and C‐peptide showed weak or no associations with CGM‐derived metrics. Importantly, insulinogenic index was inversely associated with CGM hyperglycaemic exposure and variability, indicating that lower early‐phase insulin secretion was linked to greater free‐living glucose excursions.

When examining associations between all glucose variables (CGM‐derived and clinical) and other clinical measurements, including anthropometry, blood lipids and blood pressure (Figure 3B), distinct patterns were observed. Clinical glucose and insulin variables were significantly correlated with most anthropometric measures, blood pressure and blood lipids. In particular, indices of insulin resistance (HOMA‐IR and Matsuda Index) and C‐peptide showed strong associations with BMI, body fat, blood pressure and blood lipids. In contrast, CGM‐derived glucose metrics showed few or no significant associations with these clinical measurements, indicating that they capture aspects of glucose regulation that are not reflected by anthropometry, blood lipids or blood pressure.

Notably, the association patterns were remarkably consistent between the 4‐ and 6‐year follow‐ups (Figures S2 and S3), suggesting stability in the underlying relationships between dynamic glucose regulation and clinical measures over time.

## 4. Discussion

In this longitudinal study of women with prior GDM, we examined long‐term glycaemic outcomes 4–6 years postpartum according to the diagnostic thresholds applied during pregnancy. Women diagnosed according to older GDM criteria based on higher glucose thresholds exhibited a persistently less favourable glycaemic profile, characterised by higher fasting glucose and greater free‐living glycaemic variability. Women fulfilling the lower WHO 2013 glucose thresholds represented a large at‐risk group, with a high prevalence of dysglycaemia (~50%) at follow‐up. Although CGM revealed modest differences in free‐living glycaemic patterns within this WHO‐defined group, conventional clinical measures did not differ between women with and without a GDM diagnosis during pregnancy.

These findings extend the well‐established observation that women with a history of GDM have a substantially increased risk of future dysglycaemia and T2D [1, 2]. Within this high‐risk population, however, our results demonstrate that long‐term glycaemic risk varies according to the diagnostic thresholds met during pregnancy. Given the higher pregnancy glucose concentrations in the GDM_OLD_ group, a less favourable long‐term glycaemic profile was expected. Nevertheless, several differences remained after adjustment for pregnancy glucose concentrations. In particular, fasting glucose and CGM‐derived measures of glycaemic variability and hyperglycaemic exposure remained significantly different, suggesting that the observed separation is not explained solely by the degree of hyperglycaemia during pregnancy. Because diagnostic thresholds determine which women are identified as having GDM, changes in these criteria may have important implications for identifying women at increased long‐term metabolic risk.

Following the HAPO study and the introduction of the IADPSG/WHO 2013 criteria [3–5], lower diagnostic thresholds substantially increased the number of women diagnosed with GDM. In Sweden, the CDC4G trial demonstrated increased GDM prevalence after implementation of WHO 2013 criteria but no significant reduction in large‐for‐gestational‐age births [9]. In the present long‐term follow‐up, conventional glycaemic measures at 4–6 years postpartum were largely comparable between women fulfilling WHO 2013 criteria who did and did not receive a pregnancy diagnosis and treatment. Thus, neither the primary perinatal outcomes in CDC4G nor long‐term conventional postpartum glycaemic measures in our study indicate a clear impact of treatment during pregnancy on subsequent glycaemic phenotype within the WHO‐defined group.

Nevertheless, the high prevalence of prediabetes observed among women fulfilling WHO 2013 criteria at both follow‐ups underscores that this expanded diagnostic group remains metabolically vulnerable several years after pregnancy. This is consistent with previous studies demonstrating that women meeting WHO 2013 or IADPSG criteria remain at increased risk of later dysglycaemia and T2D [1, 6, 27]. Given that progression to T2D is associated with an increased risk of long‐term complications, including cardiovascular and microvascular disease, early identification and monitoring of dysglycaemia in this population are clinically important. These findings support continued postpartum surveillance in women with prior GDM, irrespective of the diagnostic threshold applied during pregnancy.

Although conventional clinical measures did not differ between GDM_WHO_− and GDM_WHO_+, CGM identified modest differences in daily glucose dynamics. These differences were small in magnitude and remained largely within normoglycaemic ranges and therefore do not suggest a clear long‐term effect of pregnancy treatment on overall glycaemic status. However, these findings suggest that CGM may detect subtle differences in free‐living glucose regulation that are not captured by conventional clinical measurements, although the clinical significance of these modest differences remains uncertain.

The most consistent finding was a stable separation between women diagnosed according to older, higher glucose threshold criteria and those fulfilling the lower WHO 2013 thresholds. Differences in fasting glucose and CGM‐derived variability persisted after adjustment for pregnancy glucose levels, indicating that the separation was not solely explained by pregnancy glycaemia. In contrast, differences in postload OGTT measures and HbA1c were substantially attenuated after such adjustment. This suggests that part of the separation in conventional measures reflects underlying pregnancy glycaemia, whereas CGM‐derived variability and hyperglycaemic exposure capture aspects of long‐term glucose regulation not fully accounted for by pregnancy glycaemia. Importantly, these differences were observed despite similar BMI, body composition and HOMA‐IR, as well as greater weight reduction in the GDM_OLD_ group, indicating that overall adiposity and fasting insulin resistance do not fully explain the long‐term divergence. The inverse association between insulinogenic index and CGM‐derived hyperglycaemic exposure and variability (i.e., lower early insulin response was associated with higher glucose levels and greater variability) supports a role for impaired early‐phase insulin secretion in determining free‐living glycaemic patterns. This is consistent with current models of GDM pathophysiology and postpartum progression [16, 28, 29].

Our findings are consistent with our previous 2‐year follow‐up of this cohort, in which women diagnosed using older criteria displayed a more adverse metabolic profile despite similar BMI [18]. The present study demonstrates that this separation persists to 4–6 years postpartum and is evident not only in OGTT‐ and HbA1c‐based measures but also in CGM‐derived measures of glycaemic variability, which have been proposed as sensitive markers of early dysglycaemia [14, 15, 17].

CGM‐derived metrics were most strongly related to glucose‐based measures, including OGTT glucose and HbA1c, while showing limited associations with anthropometric measures, blood lipids and blood pressure. In contrast, conventional clinical glucose variables were more closely related to these cardiometabolic risk markers. Together, these findings suggest that CGM captures aspects of day‐to‐day glucose regulation, particularly postprandial excursions and glycaemic variability, that complement rather than replace conventional clinical measurements. The consistent correlation patterns observed at 4 and 6 years indicate that these relationships reflect stable features of glucose regulation rather than short‐term variation.

From a clinical perspective, our findings highlight the importance of sustained postpartum follow‐up in women with prior GDM, irrespective of the diagnostic threshold applied during pregnancy. Although women diagnosed according to older, higher glucose thresholds appear to carry the most pronounced long‐term glycaemic burden, a substantial proportion of women fulfilling WHO 2013 criteria exhibited prediabetes several years postpartum, supporting that this broader group remains clinically relevant and warrants follow‐up. Importantly, several cases of T2D were identified at the study visits that had not previously been diagnosed in routine follow‐up. Only a minority of T2D cases had been recognised before study assessment in routine care, indicating that clinically relevant dysglycaemia may remain undetected several years after GDM. Our observations suggest that CGM may complement conventional postpartum assessment by characterising free‐living glucose regulation and glycaemic variability. However, the present findings do not establish that CGM improves clinical risk prediction beyond conventional measures such as OGTT and HbA1c. Future longitudinal studies should therefore determine whether CGM‐derived metrics provide incremental prognostic value before CGM can be recommended for routine postpartum follow‐up.

### 4.1. Strengths and Limitations

Strengths of this study include the long follow‐up duration, repeated assessments, detailed OGTT phenotyping and the use of CGM to characterise free‐living glycaemia. The opportunity to compare groups defined by different pregnancy glucose thresholds within the same cohort provides a unique perspective on the long‐term implications of changing diagnostic GDM criteria.

Limitations include potential selection bias related to participation in postpartum follow‐up, which may have led to underestimation of dysglycaemia prevalence, as women at highest risk are often less likely to attend follow‐up [30, 31]. The COVID‐19 pandemic may also have influenced lifestyle behaviours during the study period. However, as the pandemic preceded the 4‐ to 6‐year follow‐ups and would likely have affected all groups similarly, it is unlikely to explain the observed between‐group differences.

## 5. Conclusions

Long‐term glycaemic outcomes 4–6 years postpartum differ according to the glucose thresholds used to diagnose GDM. Women diagnosed according to older, higher glucose thresholds continue to carry a greater glycaemic burden, with higher fasting glucose and greater free‐living glycaemic variability. Women fulfilling WHO 2013 criteria represent a broad at‐risk group, with a high prevalence of prediabetes several years after pregnancy. Within this WHO‐defined group, CGM detected differences, albeit modest, in daily glucose patterns not captured by conventional clinical measures. These findings support sustained postpartum follow‐up after GDM and suggest that CGM may serve as a complementary tool for characterising free‐living glucose regulation, whereas its role in improving clinical risk prediction remains to be established.

## Author Contributions

Conceptualization: U.A‐H., V.S., K.W. and A.H. Methodology: U.A‐H., E.K. and A.H. Formal analysis and data curation: U.A‐H., E.K. and M.T. Writing—original draft preparation: U.A‐H., M.T. and E.K. Writing—review and editing: U.A‐H., E.K., K.W., V.S., S.Å. and A.H. Visualization: U.A‐H., M.T. and E.K. Project administration: A.H.

## Funding

Financial support for the study was provided by the Emil and Wera Cornell Foundation, the Tore Nilson Foundation, the Erik and Lily Philipsons Foundation and the Swedish state through the ALF agreement between the Swedish government and the county councils (ALFGBG‐720851 and ALFGBG‐970689).

## Disclosure

All authors have read and approved the final version of the manuscript. The corresponding author had full access to all of the data in this study and takes complete responsibility for the integrity of the data and the accuracy of the data analysis.

## Conflicts of Interest

The authors declare no conflicts of interest.

## Supporting information


**Supporting Information** Additional supporting information can be found online in the Supporting Information section. Table S1: Extended comparison for clinical and CGM‐derived variables at 4 and 6 years postpartum in women meeting older diagnostic criteria (GDM_OLD_) and WHO 2013 criteria (GDM_WHO_). Table S2: Extended comparison for clinical and CGM‐derived variables at 4 and 6 years postpartum in women fulfilling WHO 2013 criteria, comparing those without a clinical diagnosis or treatment during pregnancy (GDM_WHO_−) and those with diagnosis and treatment (GDM_WHO_+). Figure S1: Glucose tolerance categories and OGTT profiles in women fulfilling WHO 2013 criteria with no clinical diagnosis (GDM_WHO_−) or with diagnosis and treatment (GDM_WHO_+). (A,D) Distribution of glucose tolerance categories at 4 and 6 years postpartum. (B–C) OGTT glucose and insulin curves at 4 years postpartum. (E–F) OGTT glucose and insulin curves at 6 years postpartum. Values are presented as mean ± SE. Group differences in glucose tolerance distribution were assessed using *χ* ^2^ tests and differences in glucose curves using comparisons of glucose area under the curve (AUC). There were no significant between‐group differences at specific time points for either glucose or insulin. Figure S2: Heatmap of associations between clinical glucose variables and CGM‐derived metrics at 4 years postpartum. Associations were assessed using Spearman correlation coefficients. Colour intensity represents the magnitude and direction of Spearman′s rho (*ρ*). Asterisks denote statistical significance ( ^∗^ *p* < 0.05,  ^∗∗^ *p* < 0.01,  ^∗∗∗^ *p* < 0.001). Figure S3: Heatmap of associations between clinical glucose variables and CGM‐derived metrics at 6 years postpartum. Associations were assessed using Spearman correlation coefficients. Colour intensity represents the magnitude and direction of Spearman′s rho (*ρ*). Asterisks denote statistical significance ( ^∗^ *p* < 0.05,  ^∗∗^ *p* < 0.01,  ^∗∗∗^ *p* < 0.001).

## Data Availability

The datasets analysed in this study contain sensitive personal information and cannot be shared publicly due to legal restrictions under the Swedish Public Access to Information and Secrecy Act (SFS 2009:400). Data may be made available upon reasonable request to the University of Gothenburg (contact: administrative lawyer Katarina Nyström, katarina.nystrom@gu.se) or to the corresponding author.
