# Supplementary material for: Postpartum Glycaemic Phenotype and Continuous Glucose Monitoring at 4–6 Years After Gestational Diabetes Defined by Different Diagnostic Criteria
Source: J Diabetes Res. 2026 Sep 8;2026:7103883. doi: 10.1155/jdr/7103883 (PMC13552220; doi:10.1155/jdr/7103883)
Supplement: Supplementary file 1 [file JDR-2026-7103883-s001.docx]

**Supplement**

***Table S1.*** *Extended comparison for clinical and CGM-derived variables at 4 and 6 years postpartum in women meeting older diagnostic criteria (GDM_OLD_) and WHO 2013 criteria (GDM_WHO_).*

|  | **4 years postpartum** | | | | **6 years postpartum** | | | | **Overall** | |
| --- | --- | --- | --- | --- | --- | --- | --- | --- | --- | --- |
|  | **Control** | **GDM_OLD_** | **GDM_WHO_** | **P raw** | **Control** | **GDM_OLD_** | **GDM_WHO_** | **P raw** | **P** | **Padj** |
| BMI | 22.0 ± 1.6 | 28.3 ± 6.5 | 29.3 ± 7.2 | 0,249 | 22.4 ± 1.9 | 28.4 ± 6.6 | 29.1 ± 6.9 | 0,468 | **0.018** | 0.343 |
| Body Fat % | 23.6 ± 6.0 | 39.0 ± 9.7 | 39.4 ± 9.8 | 0,803 | 26.6 ± 6.1 | 39.2 ± 9.2 | 39.3 ± 9.5 | 0,978 | 0.557 | 0.188 |
| Weight retention (kg)* | -1.3 ± 2.1 | -0.8 ± 6.8 | 2.1 ± 8.7 | **0.003** | 0.4 ± 3.7 | 0.5 ± 7.6 | 2.0 ± 9.0 | 0.159 | **0.016** | 0.408 |
| Waist (cm) | 71.8 ± 3.9 | 92.7 ± 13.9 | 95.0 ± 16.0 | 0,221 | 73.6 ± 5.1 | 92.9 ± 14.8 | 94.3 ± 16.3 | 0,472 | 0.164 | 0.384 |
| HDL (mmol/L) | 2.1 ± 0.4 | 1.3 ± 0.3 | 1.3 ± 0.3 | 0,559 | 1.8 ± 0.4 | 1.4 ± 0.4 | 1.4 ± 0.3 | 0,963 | 0.813 | 0.658 |
| LDL (mmol/L) | 3.2 ± 0.8 | 3.1 ± 0.8 | 3.1 ± 0.8 | 0,729 | 2.8 ± 0.8 | 3.3 ± 0.8 | 3.2 ± 0.8 | 0,619 | 0.981 | 0.782 |
| TG (mmol/L) | 0.81 ± 0.21 | 1.1 ± 0.5 | 1.1 ± 0.8 | 0,545 | 0.72 ± 0.30 | 1.0 ± 0.5 | 1.0 ± 0.7 | 0,994 | 0.410 | 0.499 |
| Diastolic BP (mm Hg) | 71 ± 7 | 79 ± 11 | 77 ± 10 | 0,139 | 75.0 ± 8.1 | 78.3 ± 10.4 | 77.5 ± 9.7 | 0,522 | 0.481 | 0.686 |
| Systolic BP (mm Hg) | 104 ± 9 | 114 ± 14 | 112 ± 13 | 0,197 | 112 ± 9 | 114 ± 15 | 114 ± 14 | 0,981 | 0.4390 | 0.412 |
| HbA1c (mmol/mol) | 32.2 ± 1.9 | 35.9 ± 5.8 | 33.5 ± 2.9 | **<0,001** | 31.4 ± 2.9 | 35.9 ± 4.4 | 33.8 ± 3.6 | **<0,001** | **<0.001** | 0.172 |
| Fasting Glucose (mmol/L) | 5.1 ± 0.5 | 5.8 ± 0.8 | 5.5 ± 0.5 | **<0,001** | 5.2 ± 0.4 | 5.7 ± 0.9 | 5.5 ± 0.5 | **0,021** | **<0.001** | **0.039** |
| 2h Glucose (mmol/L) | - | 7.2 ± 2.6 | 5.8 ± 1.3 | **<0.001** |  | 6.7 ± 2.2 | 5.7 ± 1.4 | **0,001** | **<0.001** | **0.048** |
| HOMA-IR | 1.2 ± 0.6 | 2.3 ± 2.0 | 2.1 ± 1.3 | 0,385 | 1.0 ± 0.4 | 2.0 ± 1.5 | 1.8 ± 1.4 | 0,502 | 0.626 | 0.767 |
| Fasting insulin (mU/L) | 5.1 ± 2.0 | 8.3 ± 6.0 | 8.2 ± 4.9 | 0.862 | 4.3 ± 1.3 | 7.6 ± 5.2 | 7.4 ± 5.4 | 0.805 | 0.998 | 0.953 |
| 2h Insulin (mU/L) | - | 43.9 ± 44.7 | 30.8 ± 25.3 | **0.023** | - | 41.7 ± 40.3 | 33.9 ± 33.4 | 0.148 | 0.292 | 0.950 |
| Insulinogenic Index | - | 1.00 ± 2.35 | 1.04 ± 1.81 | 0.915 | - | 0.54 ± 0.60 | 0.99 ± 2.1 | **0.011** | 0.326 | 0.524 |
| Matsuda Index | - | 6.71 ± 3.73 | 6.94 ± 4.20 | 0.703 | - | 6.63 ± 3.86 | 8.66 ± 8.99 | **0.015** | 0.149 | 0.086 |
| *CGM metrics* |  |  |  |  |  |  |  |  |  |  |
| Mean Glucose (mmol/L) | - | 5.9 ± 0.9 | 5.5 ± 0.5 | **0,002** | - | 5.7 ± 0.6 | 5.5 ± 0.4 | **0,015** | **0.001** | 0.091 |
| Time in range (%) | - | 89.7 ± 14.1 | 96.4 ± 3.8 | **<0,001** | - | 92.8 ± 7.2 | 96.7 ± 4.0 | **<0,001** | **<0.001** | **0.006** |
| Time below range (%) | - | 1.7 ± 2.8 | 1.7 ± 2.8 | 0,984 | - | 1.4 ± 1.8 | 1.4 ± 2.3 | 0,895 | 0.910 | 0.458 |
| Time above range (%) | - | 8.6 ± 14.5 | 1.9 ± 3.1 | **<0,001** | - | 5.8 ± 7.3 | 1.9 ± 3.1 | **<0,001** | **<0.001** | **0.002** |
| CV Glucose | - | 17.5 ± 4.0 | 13.9 ± 2.9 | **<0,001** | - | 17.5 ± 4.0 | 14.1 ± 3.5 | **<0,001** | **<0.001** | **0.008** |
| MAGE (mmol/L) | - | 1.3 ± 0.4 | 1.1 ± 0.2 | **<0,001** | - | 1.3 ± 0.3 | 1.1 ± 0.3 | **<0,001** | **<0.001** | **0.009** |

*Values are presented as mean ± SD. P raw refers to unadjusted comparisons between GDM_OLD_ and GDM_WHO_. The control group is shown for reference and was not included in statistical comparisons. Overall P refers to longitudinal group comparison derived from linear mixed-effects models adjusted for reason for OGTT, BMI at first antenatal visit, ethnicity, and maternal age at birth. Overall Padj refer to the same model but further adjusted for pregnancy glucose values. CGM, continuous glucose monitoring; HOMA-IR, Homeostatic Model Assessment of Insulin Resistance; MAGE, mean amplitude of glycaemic excursions; CV, coefficient of variation.*Difference between follow-up weight and trimester 1 weight*

***Table S2****. Extended comparison for clinical and CGM-derived variables at 4 and 6 years postpartum in women fulfilling WHO 2013 criteria, comparing those without a clinical diagnosis or treatment during pregnancy (GDM_WHO_-) and those with diagnosis and treatment (GDM_WHO_+).*

|  | **4 years postpartum** | | | **6 years postpartum** | | | **Overall** | |
| --- | --- | --- | --- | --- | --- | --- | --- | --- |
|  | **GDM_WHO_-** | **GDM_WHO_+** | **P raw** | **GDM_WHO_-** | **GDM_WHO_+** | **P raw** | **P** | **Padj** |
| BMI | 29.3 ± 7.6 | 29.2 ± 6.7 | 0.431 | 29.1 ± 7.1 | 29.0 ± 6.6 | 0.420 | 0.451 | 0.570 |
| Body Fat % | 38.9 ± 10.1 | 40.0 ± 9.4 | 0.073 | 39.3 ± 10.1 | 39.2 ± 8.8 | 0.589 | 0.059 | 0.070 |
| Weight retention (kg)* | 1.7 ± 9.1 | 2.6 ± 8.2 | 0.460 | 2.4 ± 8.9 | 1.4 ± 8.9 | 0.435 | 0.502 | 0.619 |
| Waist (cm) | 96.1 ± 16.7 | 93.5 ± 15.0 | 0.303 | 94.4 ± 16.6 | 94.2 ± 16.0 | 0.669 | 0.777 | 0.796 |
| HDL (mmol/L) | 1.3 ± 0.3 | 1.4 ± 0.3 | 0.097 | 1.4 ± 0.3 | 1.4 ± 0.3 | 0.436 | 0.240 | 0.418 |
| LDL (mmol/L) | 3.0 ± 0.8 | 3.2 ± 0.8 | 0.170 | 3.2 ± 0.8 | 3.2 ± 0.7 | 0.888 | 0.830 | 0.644 |
| TG (mmol/L) | 1.1 ± 0.6 | 1.1 ± 0.9 | 0.170 | 1.1 ± 0.7 | 1.0 ± 0.7 | 0.550 | 0.869 | 0.869 |
| Diastolic BP (mm Hg) | 77 ± 10 | 77 ± 10 | 0.813 | 77 ± 10 | 79 ± 10 | 0.250 | 0.064 | 0.109 |
| Systolic BP (mm Hg) | 111 ± 12 | 113 ± 14 | 0.159 | 112 ± 14 | 116 ± 13 | 0.079 | **0.039** | 0.072 |
| HbA1c (mmol/mol) | 33.5 ± 2.8 | 33.4 ± 3.0 | 0.979 | 33.8 ± 3.5 | 33.9 ± 3.7 | 0.797 | 0.612 | 0.594 |
| Fasting Glucose (mmol/L) | 5.6 ± 0.5 | 5.4 ± 0.5 | 0.057 | 5.5 ± 0.5 | 5.4 ± 0.5 | 0.081 | 0.273 | 0.541 |
| 2h Glucose (mmol/L) | 5.7 ± 1.2 | 5.9 ± 1.4 | 0.301 | 5.6 ± 1.6 | 5.8 ± 1.3 | 0.257 | 0.209 | 0.175 |
| HOMA-IR | 2.0 ± 1.3 | 2.1 ± 1.3 | 0.933 | 1.9 ± 1.5 | 1.7 ± 1.3 | 0.180 | 0.519 | 0.552 |
| Fasting insulin (mU/L) | 8.2 ± 4.9 | 8.3 ± 4.8 | 0.972 | 7.7 ± 5.8 | 7.0 ± 4.8 | 0.183 | 0.420 | 0.438 |
| 2h Insulin (mU/L) | 28.9 ± 23.5 | 33.7 ± 27.6 | 0.163 | 32.1 ± 34.0 | 36.0 ± 32.7 | 0.420 | 0.242 | 0.250 |
| Insulinogenic Index | 0.98 ± 1.84 | 1.11 ± 1.78 | 0.458 | 1.16 ± 2.4 | 0.79 ± 1.8 | 0.209 | 0.384 | 0.383 |
| Matsuda Index | 7.10 ± 4.61 | 6.70 ± 3.56 | 0.406 | 9.01 ± 10.6 | 8.26 ± 6.7 | 0.705 | 0.720 | 0.766 |
| *CGM metrics* |  |  |  |  |  |  |  |  |
| Mean Glucose (mmol/L) | 5.6 ± 0.5 | 5.4 ± 0.5 | **0.007** | 5.5 ± 0.4 | 5.6 ± 0.5 | **0.010** | **<0.001** | **<0.001** |
| Time in range (%) | 96.6 ± 3.4 | 96.2 ± 4.2 | 0.522 | 97.4 ± 3.6 | 95.7 ± 4.3 | **0.004** | **0.007** | **0.045** |
| Time below range (%) | 1.4 ± 2.6 | 2.1 ± 3.2 | 0.177 | 1.2 ± 2.1 | 1.6 ± 2.6 | 0.226 | 0.125 | 0.450 |
| Time above range (%) | 1.9 ± 2.7 | 1.8 ± 3.6 | 0.640 | 1.4 ± 2.1 | 2.7 ± 3.9 | **0.005** | **0.008** | **0.013** |
| CV Glucose | 13.5 ± 3.0 | 14.6 ± 2.6 | **0.037** | 13.8 ± 3.8 | 14.5 ± 3.0 | 0.148 | **0.035** | 0.159 |
| MAGE | 1.0 ± 0.2 | 1.1 ± 0.2 | 0.732 | 1.0 ± 0.3 | 1.1 ± 0.2 | 0.051 | **0.034** | 0.122 |

*Values are presented as mean ± SD. P raw refers to unadjusted comparisons between groups. Overall P refers to longitudinal group comparison derived from linear mixed-effects models adjusted for reason for OGTT, BMI at first antenatal visit, ethnicity, and maternal age at birth. Overall Padj refer to the same model but further adjusted for pregnancy glucose values. CGM, continuous glucose monitoring; HOMA-IR, Homeostatic Model Assessment of Insulin Resistance; MAGE, mean amplitude of glycaemic excursions; CV, coefficient of variation.*Difference between follow-up weight and trimester 1 weight*

***Figure S1.* *Glucose tolerance categories and OGTT profiles in women fulfilling WHO 2013 criteria with no clinical diagnosis (GDM_WHO_-) or with diagnosis and treatment (GDM_WHO_+).*** *(A, D) Distribution of glucose tolerance categories at 4 and 6 years postpartum.
(B–C) OGTT glucose and insulin curves at 4 years postpartum.
(E–F) OGTT glucose and insulin curves at 6 years postpartum.
Values are presented as mean ± SE. Group differences in glucose tolerance distribution were assessed using χ² tests and differences in glucose curves using comparisons of glucose area under the curve (AUC). There were no significant between-group differences at specific time points for either glucose or insulin.*

***Figure S2. Heatmap of associations between clinical glucose variables and CGM-derived metrics at 4 years postpartum.***

*Associations were assessed using Spearman correlation coefficients. Colour intensity represents the magnitude and direction of Spearman’s rho (ρ). Asterisks denote statistical significance (*p < 0.05, **p < 0.01, ***p < 0.001).*

***Figure S3. Heatmap of associations between clinical glucose variables and CGM-derived metrics at 6 years postpartum.***

*Associations were assessed using Spearman correlation coefficients. Colour intensity represents the magnitude and direction of Spearman’s rho (ρ). Asterisks denote statistical significance (*p < 0.05, **p < 0.01, ***p < 0.001).*
